# Identification and Quantification of Anti-Gp.Mur Antibodies in Human Serum Using an Insect-Cell-Based System

**DOI:** 10.3390/diagnostics11060966

**Published:** 2021-05-27

**Authors:** Robert John S. Lamis, Tsong-Shi Chiueh, Chih-Hsuan Tsai, Huei-Ru Lo, Sung-Chan Wei, Yu-Chan Chao

**Affiliations:** 1 Molecular and Cell Biology, Taiwan International Graduate Program, Academia Sinica and Graduate Institute of Life Science, National Defense Medical Center, Taipei 115, Taiwan; robertjohnlamis@yahoo.com; 2 Institute of Molecular Biology, Academia Sinica, Taipei 115, Taiwan; lt22448@gmail.com (C.-H.T.); hrl@gate.sinica.edu.tw (H.-R.L.); nerv.lilith@gmail.com (S.-C.W.); 3 Graduate Institute of Life Science, National Defense Medical Center, Taipei 114, Taiwan; 4 Department of Laboratory Medicine, Linkou Chang Gung Memorial Hospital, Taipei 333, Taiwan; drche0523@gmail.com; 5 Division of Laboratory Medicine, New Taipei Municipal Tu Cheng Hospital, Tu Cheng, Taipei 236, Taiwan; 6 Department of Entomology, National Chung Hsing University, Taichung 402, Taiwan; 7 Department of Plant Pathology and Microbiology, College of Bioresources and Agriculture, National Taiwan University, Taipei 106, Taiwan

**Keywords:** baculovirus, blood banking, cell-based assay, Gp.Mur, hemagglutination inhibition

## Abstract

Gp.Mur is a clinically relevant antigen of the MNS blood group system that is highly prevalent in several Asian populations. Its corresponding antibody, anti-Gp.Mur, has been implicated in hemolytic transfusion reactions and hemolytic disease of the fetus and newborn. Currently, identifying and confirming anti-Gp.Mur antibody presence in sera via agglutination of a panel of red blood cells (RBCs) is inefficient and difficult to quantify. Using a baculovirus expression system to express Gp.Mur antigen on insect cell surfaces, we have developed a quantitative cell-based system to confirm the presence of anti-Gp.Mur antibody in human serum. We obtained 10 serum samples preidentified as having anti-Gp.Mur antibody and another 4 samples containing noncorresponding antibodies from hospital patients. Insect cells displaying Gp.Mur antigen successfully adsorbed anti-Gp.Mur antibody in the sera and inhibited the RBC agglutination mediated by this antibody. By varying the concentration of Gp.Mur-displaying cells, we could grade levels of RBC agglutination by anti-Gp.Mur antibody. Densitometric analysis further enabled quantitative determinations of hemagglutination inhibition by Gp.Mur-displaying cells. We believe that this cell-based hemagglutination inhibition system greatly improves or supplements existing technology and is a convenient means for accurately identifying and quantifying anti-Gp.Mur antibody.

## 1. Introduction

Gp.Mur antigen, previously known as Miltenberger antigen subtype III (Mi.III) or Mi^a^ antigen, is one of several antigens of the MNS blood group system [1,2]. Gp.Mur antigen is a variant of the red blood cell (RBC) glycophorins in the MNS system, and it most likely evolved from crossover events between glycophorin A and glycophorin B [3,4]. In Southeast and South Asian human populations, including Taiwan, incidence of Gp.Mur antigen is higher (ranging from 2% to 7%) relative to white Caucasian (0.0098%) and Japanese (0.0068%) populations, making it one of the most clinically relevant antigens for blood transfusions in those regions [5,6,7,8,9]. Anti-Gp.Mur antibody is one of the most commonly identified RBC alloantibodies among thalassemia and multitransfused patients in Taiwan [10,11]. An aboriginal group in Taiwan, the Ami tribe, presents a markedly high incidence of this antigen (up to 88.4%), which has been credited to their athleticism [9,12]. Furthermore, the Yami and Puyuma tribes exhibit Gp.Mur antigen frequencies of 34.3% and 21.2%, respectively, so anti-Gp.Mur antibody may occur naturally in these ethnic groups [5]. Given its clinical relevance, screening for the anti-Gp.Mur antibody is routinely conducted in Taiwan on patients who are about to receive a blood transfusion [13]. Anti-Gp.Mur antibody is also one of the most frequently reported RBC antibodies in Southeast and East Asia, probably due to the high frequency of Gp.Mur antigen there and consequent alloimmunization [5,8]. Anti-Gp.Mur antibody is clinically significant as it has been implicated in hemolytic disease of the fetus and newborn (HDFN) [14,15] and hemolytic transfusion reactions (HTR) [6]. HTR can be abrogated if Gp.Mur antigen-negative RBCs are transfused into patients having the anti-Gp.Mur antibody [16]. Because anti-Gp.Mur antibody may cause adverse reactions if not identified in patients before receiving blood transfusion, screening and identification of anti-Gp.Mur antibody is crucial, especially in situations where alloimunization of Gp.Mur antigen is possible.

Screening and identification of antibodies of concern, such as the anti-Gp.Mur antibody, require the use of a panel of reagent RBCs that has been phenotyped for the most relevant antigens [17]. Given that hundreds of antigens are expressed by RBCs, inaccurate results may occur. To aid the identification of antibodies of concern, clinically relevant RBC antigens may be expressed individually as soluble recombinant proteins [18,19,20,21,22,23]. Using such soluble recombinant proteins in hemagglutination inhibition assays has proven successful in confirming or excluding the presence of the corresponding antibody [18,19,20,21,22,23]. However, these antigens are transmembrane proteins and some are even multitransmembrane proteins, so they may not be able to maintain their correct epitope conformations if expressed in soluble form. Furthermore, the purification of membrane proteins is tedious, and the hydrophobic membrane domain may aggregate after purification. We propose to resolve these crucial issues by displaying these RBC antigens on a cell line to preserve their conformation and use the antigen-displaying cells as adsorbing material.

Of the many expression systems available, the baculovirus–insect cell expression system may represent an ideal heterologous system for generating antigen-displaying cells applicable to cell-based hemagglutination inhibition assays. Baculoviruses are insect viruses, and the species *Autographa californica* multiple nucleopolyhedrovirus (AcMNPV) has been widely used to produce eukaryotic recombinant proteins, such as human acid sphingomyelinase and human interferon-γ, with antiviral activity [24,25]. AcMNPV can integrate a large insertion of foreign DNA, and insect cells infected by the recombinant virus can express a foreign protein with extensive post-translational modifications and proper protein oligomerization, which cannot be achieved using bacterial expression systems [26,27,28]. By fusing the foreign protein with GP64, the surface glycoprotein of AcMNPV, a foreign protein can be displayed on the plasma membrane of insect cells [29]. This surface display technology has been used to express several membrane, secretory, or cytosolic proteins, enabling assays of protein function or interacting partners [29,30,31,32,33]. When assaying a target protein that is of mammalian origin, the insect cell platform may reduce interference caused by intrinsic proteins in mammalian cell systems, and the strong baculovirus promoters (e.g., *p10* or *polh*) result in high expression of the target foreign proteins [34,35,36]. In addition, because baculovirus blocks the transcription and translation of host cells, it further reduces nonspecific background signal of antibody/antigen interactions [37].

In this study, we established an insect-cell-based hemagglutination inhibition assay for Gp.Mur antigen (Supplementary Figure S1). This system enables quantitative assessment of serum antibody adsorption by Gp.Mur antigen, representing a significant advance over conventional alloantibody typing using RBCs or purified antigens. By varying the concentration of adsorbing cells, we could quantify their degree of hemagglutination inhibition. Inhibition could be observed by conventional visual grading or graded quantitatively by precision densitometry. Furthermore, we performed a stability assay and found that insect cells displaying Gp.Mur antigen maintained their inhibitory activity despite long-term storage. Not only does our technology aid in accurately confirming anti-Gp.Mur antibody presence in human sera but its further development could also assist in identifying other clinically significant alloantibodies from other blood group systems.

## 2. Materials and Methods

### 2.1. Cell Cultures and Viruses

*Spodoptera frugiperda* IPLB-Sf21 (Sf21) cells were cultured at 26 °C in TC100 insect medium (Thermo Fisher Scientific, Waltham, MA, USA) with 10% fetal bovine serum (FBS). *Trichoplusia ni* BTI-TN-5B1-4 (Hi5) cells were cultured at 26 °C in ESF 921 serum-free insect cell culture medium (Expression Systems, LLC, CA, USA) without adding FBS. Recombinant baculovirus was generated using FlashBAC™ baculovirus genome (Mirus, Madison, WI, USA) and propagated in Sf21 cells.

### 2.2. Generation of Baculovirus Expression Construct

The transfer vector [38] was constructed using pBacPAK8 (Clontech, Laboratories Inc., Fremont, CA, USA), with its polyhedrin promoter replaced with the *TriEx* promoter from pTriEx™-3 vector (Millipore, Burlington, MA, USA). A *td-tomato* reporter gene driven by an internal ribosome entry site (IRES) from *Rhopalosiphum padi* virus (RhPV-IRES) [39] was inserted into the vector to act as the fluorescence reporter. A nucleotide sequence encoding full-length Gp.Mur were synthesized according to GenBank accession number AF090739.1 from the National Center of Biotechnology Information (NCBI). Gp.Mur sequence corresponding to amino acids 1–122 was amplified by polymerase chain reaction (PCR) and subcloned into baculovirus transfer vector harboring the GP64 cytoplasmic tail domain (6C) along with a honeybee melittin signal peptide and a hexameric histidine tag (6H) at the N-terminal of Gp.Mur. The expression construct was generated using In-Fusion^®^ HD cloning kit (Clonetech, Laboratories Inc., Fremont, CA, USA) according to the manufacturer’s manual. The Gp.Mur construct was cotransfected with FlashBAC™ baculovirus genome in SF21 cells to generate a recombinant baculovirus expressing Gp.Mur antigen.

### 2.3. Western Blot Analysis

The recombinant Gp.Mur construct was expressed in Hi5 cells by infecting cells with the recombinant baculovirus expressing Gp.Mur antigen using a multiplicity of infection of 1 (MOI = 1). After incubation at 26 °C at different time points, the cells were detached, and the culture medium was removed by low-speed centrifugation. Dulbecco’s phosphate-buffered saline (DPBS) was added to remove the remaining culture medium and to wash the cells. The harvested cells were lysed using RIPA lysis and extraction buffer (Thermo Fisher Scientific, Waltham, MA, USA), and the cell lysates were then separated on a 10% sodium dodecyl sulfate–polyacrylamide gel (SDS-PAGE; Omic Bio, Taipei, Taiwan). Proteins on gels were transferred to a polyvinylidene fluoride (PVDF) membrane and Western blotted using mouse anti-His antibody (1:5000, GeneTex GTX628914; GeneTex Inc., CA, USA). As negative control, cells infected with wild-type baculovirus harboring the reporter gene alone (WT-td) was used simultaneously in the assays. As a loading control, glyceraldehyde 3-phosphate dehydrogenase (GAPDH) expression was determined using rabbit anti-GAPDH (1:5000, GeneTex GTX100118; GeneTex Inc., CA, USA).

### 2.4. Cell-Based Enzyme-Linked Immunosorbent Assay (ELISA)

Hi5 cells (1 × 10^4^) were seeded onto a 96-well plate (Falcon) and infected with recombinant baculovirus expressing Gp.Mur antigen using MOI = 1. At 3 days post infection (dpi), the cells were fixed with 4% paraformaldehyde and blocked with 3% bovine serum albumin (BSA) in DPBS for 1 h. The cells were then incubated for 2 h with mouse anti-His antibody (1:5000, GeneTex GTX628914) at 25 °C. After three washes with DPBS with 0.1% Tween^®^ 20 (Sigma-Aldrich, CO, USA) (DPBST), HRP-conjugated goat anti-mouse IgG secondary antibody was added (1:5000) and incubated for 1 h. The enzymatic reaction with 3,3′,5,5′-tetramethylbenzidine (TMB) substrate was stopped by adding 2N H_2_SO_4_ after 5 min of color development. The optical density (OD) of the Gp.Mur antigen expressed in insect cells was compared to that of WT-td-infected cells and uninfected cells (CO) to quantify surface expression of Gp.Mur antigen (EnSpire Series Multilabel Plate Reader, PerkinElmer).

### 2.5. Human Serum Samples

Human serum samples preidentified as having anti-Gp.Mur antibody by means of the polybrene method [40] were obtained from Linkou Changung Memorial Hospital, Taipei, Taiwan. The sera were blood bank samples that had undergone routine antibody identification as part of the antibody screening process for blood transfusion. Samples deemed positive for anti-Gp.Mur antibody were stored for future research use and included in the current study. Likewise, serum samples positive for antibodies for other blood groups, namely anti-Le^b^, anti-E, anti-D, and anti-S, were obtained to serve as controls. Upon receipt, the samples were re-tested for anti-Gp.Mur or antibodies of other blood groups using reagent RBCs containing the corresponding antigens (MeDiPro Antibody Screening Cell, Formosa, Biomedical Technology Corp., Taipei, Taiwan). The tests were performed separately on the immediate spin (IS) phase (at room temperature) and the antihuman globulin (AHG) phase (at 37 °C) [17].

All experimental procedures were approved by the Ethics Committees of Linkou Changung Memorial Hospital (IRB No. 201800282B0), Taiwan, and performed in line with the Declaration of Helsinki II principles. Written informed consent was obtained from all participants prior to enrollment in the study.

### 2.6. Preparation of Insect Cell Suspension

For hemagglutination inhibition assay using cell surface display of Gp.Mur antigen, Hi5 cells were infected with the recombinant baculovirus using MOI = 1 in a T150 cell culture flask. As a control, the WT-td baculovirus was used to infect the cells. At 3 dpi, the cells were detached, pelleted down, and the culture medium was removed. After washing twice with DPBS, cell numbers were counted before resuspending them in DPBS to a concentration of 5.0 × 10^6^ cells/mL (Supplementary Figure S1). The cell suspension was then stored at 4 °C until use.

### 2.7. Hemagglutination Inhibition Assay

To perform the hemagglutination inhibition assay, varying amounts of insect cell suspension resulting in different cell concentrations (1.0 × 10^5^, 5.0 × 10^4^, and 2.5 × 10^4^) were pipetted into separate microtubes containing 100 μL of human serum preidentified as harboring anti-Gp.Mur antibody (Supplementary Figure S1). The insect cell–serum suspension was agitated in a mechanical rotator at 1000 rpm for 25 min, after which it was centrifuged at 10,000 rpm for 5 min to separate the adsorbed serum from the insect cells. The adsorbed serum was then transferred to a separate test tube, and 50 μL of indicator RBC expressing Gp.Mur antigen (MeDiPro Antibody Screening Cell, Formosa, Biomedical Technology Corp., Taipei, Taiwan) was added. The RBC–serum suspension was then centrifuged for 30 s at 800 × *g*. After centrifugation, the cell pellet was gently dislodged and graded for agglutination [17]. For each serum sample, adsorption and hemagglutination inhibition were performed using the phase in which the serum sample showed greatest reactivity in the preliminary test, i.e., samples A–I were adsorbed at room temperature and the RBC agglutination test was done in the IS phase, whereas sample J was adsorbed at 37 °C and the agglutination test was done in the AHG phase. Presence of anti-Gp.Mur antibody was confirmed when serum samples adsorbed on cells displaying Gp.Mur antigen on their surfaces displayed agglutination inhibition or a reduction in the grade of agglutination relative to agglutination by WT-td-infected cells. Photographs of all test tubes used in agglutination assays were taken. To visualize agglutinates under microscopy and to quantify agglutination by means of densitometry, RBC agglutinates were carefully pipetted into 96-well plates and microscopic images were taken. The 96-well plates were placed into a Amersham^TM^ Tyhoon 5 Imager (GE Healthcare, IL, USA) imager to conduct densitometric scans of the agglutinates. The density of the agglutinates as reflected in the scans was quantified using ImageQuant TL 8.2 software (GE, Healthcare, Il, USA) and expressed as signal intensity. The intensity of the agglutinates of the serum samples adsorbed by Gp.Mur-displaying insect cells was compared with that of the WT-td and expressed as % inhibition. To determine the specificity of hemagglutination inhibition, serum samples containing antibodies for other blood groups were used in the assay.

### 2.8. Stability Assay of Cells Displaying Gp.Mur Antigen

To determine the stability of Gp.Mur antigen expressed in insect cells, we performed hemagglutination inhibition assays as described above on the same serum samples when the cells had been freshly prepared and after the cells had been stored at 4 °C for 30 days.

### 2.9. Calculations and Statistical Analyses

For cell-based ELISA and stability assays, triplicate values were taken and error bars represent the standard deviation of the mean. For hemagglutination inhibition assay, duplicates for each level of treatment were obtained for each serum sample. For quantitative densitometry data, the values represent the average of duplicates and statistical bars represent the range of the values. To compare methods, Pearson’s *r* was calculated in Prism software. To calculate the % inhibition, the visual grading and densitometry reading of the insect cells with the expressed Gp.Mur antigen was subtracted from the reading of the WT-td, and the resulting value was then divided by the reading of the WT-td and multiplied by 100.

## 3. Results

### 3.1. Construction of Recombinant Baculovirus That Can Display Gp.Mur Antigen on Its Cell Surface

**Figure 1:**
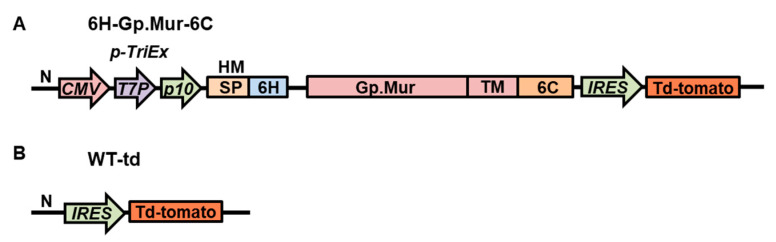
Construction of recombinant baculovirus expressing Gp.Mur antigen. (**A**) 6H-Gp.Mur-6C encodes the Gp.Mur extracellular domain and its transmembrane domain (TM) fused with the 6C. (**B**) Wild-type baculovirus with the reporter gene alone (WT-td). The 6H-Gp.Mur-6C construction is fused to hexameric histidine tag (6H). *p-TriEx*: composite promoter; *CMV*: mammalian *CMVie* promoter; *T7P*: bacterial *T7* promoter; *p10*: baculovirus *p10* promoter; HM: honeybee melittin; SP: signal peptide; *IRES*: *Rhopalosiphum padi* virus internal ribosomal entry site; Td-tomato: *td-tomato* reporter gene.

To display Gp.Mur antigen on insect cell surfaces, we generated a construct that encodes the Gp.Mur extracellular domain and its transmembrane domain fused with the AcMNPV 6C (6H-Gp.Mur-6C; Figure 1A). For control, we generated another recombinant baculovirus construct containing only the red fluorescence reporter gene, (*td-tomato*) wild-type-td (WT-td; Figure 1B). Expression of the construct was driven by a composite *TriEx* promoter, which is a combination of mammalian (*CMVie*), bacterial (*T7*), and baculovirus (*p10*) promoters. We fused a honeybee melittin signal peptide to the N-terminal of the Gp.Mur construct to enable extracellular secretion of the recombinant proteins. We assumed the recombinant protein would be anchored on the plasma membrane of the insect cell membrane via the transmembrane domain of Gp.Mur antigen fused with the 6C. A *td-tomato* fluorescence gene was inserted downstream of the Gp.Mur construct, which was driven by an IRES sequence. The *td-tomato* fluorescence signal therefore reflected successful baculovirus infection of cells and successful expression of Gp.Mur recombinant protein. After successful cotransfection of the Gp.Mur construct with the baculovirus genome, the resulting recombinant baculovirus harboring the construct was named v6H-Gp.Mur-6C.

### 3.2. Expression and Surface Display of Gp.Mur Antigen by Recombinant Baculovirus in Insect Cells

**Figure 2:**
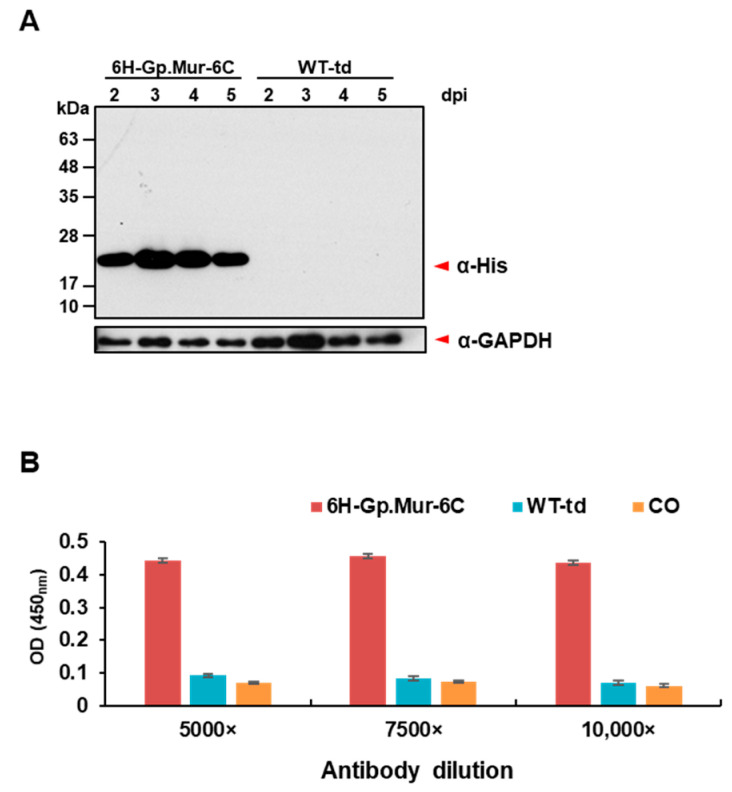
Cell surface expression of the Gp.Mur construct. (**A**) Protein expression levels of recombinant baculovirus v6H-Gp.Mur-6C-infected Hi5 cells at different time points by Western analysis. (**B**) Protein expression levels of Gp.Mur recombinant baculovirus v6H-Gp.Mur-6C-infected Hi5 cells using cell-based ELISA. Primary antibody used in Western analysis is mouse anti-His antibody (α-His, 1:5000), for internal control, rabbit anti-GAPDH, (α-GAPDH, 1:5000) was used; primary antibody used in cell-based ELISA is anti-His antibody at different dilutions. DPI: days post infection; WT-td: WT-td-infected Hi5 cells; CO: cell only (uninfected cells).

To determine if the recombinant baculovirus could express Gp.Mur antigen in Hi5 cells, we infected the cells at different time points. Cells were lysed, and Gp.Mur expression was detected by Western blot analysis using anti-His antibody (Figure 2A). The data showed that the optimum time point of protein expression was 3 dpi. To better estimate the levels of Gp.Mur surface display in Hi5 cells, we fixed the infected Hi5 cells in the wells of a 96-well cell culture plate and determined antigen–anti-His antibody interactions by means of ELISA. This cell-based ELISA revealed that 6H-Gp.Mur-6C exhibited substantial protein expression relative to WT-td and CO (Figure 2B).

### 3.3. Hemagglutination Inhibition Assay on Human Serum Samples Harboring Anti-Gp.Mur Antibody

#### 3.3.1. Characterization of the Serum Samples

Samples obtained from the blood bank of Linkou Changung Memorial Hospital, preidentified as containing anti-Gp.Mur antibody, were validated as having that antibody at the IS and AHG phases using reagent RBCs expressing Gp.Mur (MeDiPro Antibody Screening Cell). Most of the serum samples presented a strong hemagglutination reaction at the IS phase (grades ranging from 3+ to 4+), except for sample J (grade 2+) (Table 1). Sample J exhibited a higher grade of 3+ at the AHG phase, perhaps because it had been exposed to Gp.Mur antigen more than once [41].

#### 3.3.2. Adsorption Using Gp.Mur Antigen Expressed in Insect Cells

Because RBCs host multiple antigens, a positive hemagglutination result means that the serum sample contains antibody or antibodies to at least one of the many antigens on the surface of the RBCs. Suspected antibody identity must be confirmed by further phenotyping a patient for the absence of corresponding RBC antigens or by reacting the serum with another batch of reagent RBCs that host the corresponding antigens. However, if a suspected antibody is adsorbed using a specifically expressed antigen and then subjected to an RBC hemagglutination assay, that would directly confirm antibody identity. Here, we expressed Gp.Mur antigen on the surface of insect cells, acting as the adsorbing material, to confirm the presence of anti-Gp.Mur antibody in serum samples. Varying concentrations of insect cells were added to serum samples and then indicator RBCs containing the target antigen were added (Supplementary Figure S1). Resulting RBC hemagglutination was visually graded [17].

#### 3.3.3. Grading of Hemagglutination

**Figure 3:**
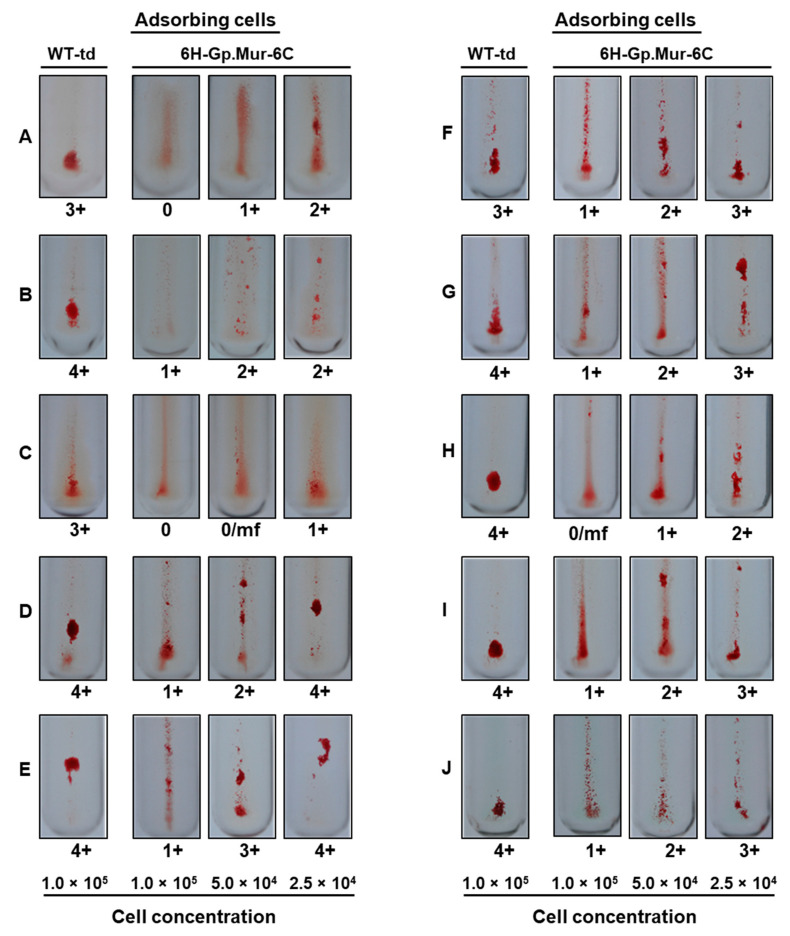
Hemagglutination inhibition based on the conventional tube method. A total of 10 serum samples (**A**–**J**) were treated with varying concentrations of Hi5 cells expressing Gp.Mur antigen (1.0 × 10^5^, 5.0 × 10^4^, or 2.5 × 10^4^). As a control, we used WT-td-infected Hi5 cells (1.0 × 10^5^). Evaluation of levels of hemagglutination revealed a dose-dependent hemagglutination inhibitory response due to anti-Gp.Mur antibody in the sera. Samples (**A**–**I**) were tested at the IS phase, whereas sample (**J**) was tested at the AHG phase. The 0/mf indicates presence of small agglutinates among nonagglutinated cells.

To determine if adsorption of anti-Gp.Mur antibody by Gp.Mur antigen expressed in insect cells inhibited hemagglutination, we first employed the conventional grading system that ranks hemagglutination on a scale of 0 (no agglutination) to 4+ (a single solid agglutinate) [17]. This approach revealed varying degrees of hemagglutination among samples, though all 10 samples presented inhibition relative to WT-td cells. Hemagglutination inhibition assays were performed at the IS phase for samples A–I, whereas it was performed at the AHG phase for sample J. We observed a dose–response inhibitory effect (Figure 3), with the highest cell concentration (1.0 × 10^5^) resulting in almost complete inhibition of hemagglutination (Figure 3; samples A, C, and H). However, this system of grading is highly subjective, especially when evaluating lower grades of agglutination.

#### 3.3.4. Quantitative Grading of Hemagglutination

**Figure 4:**
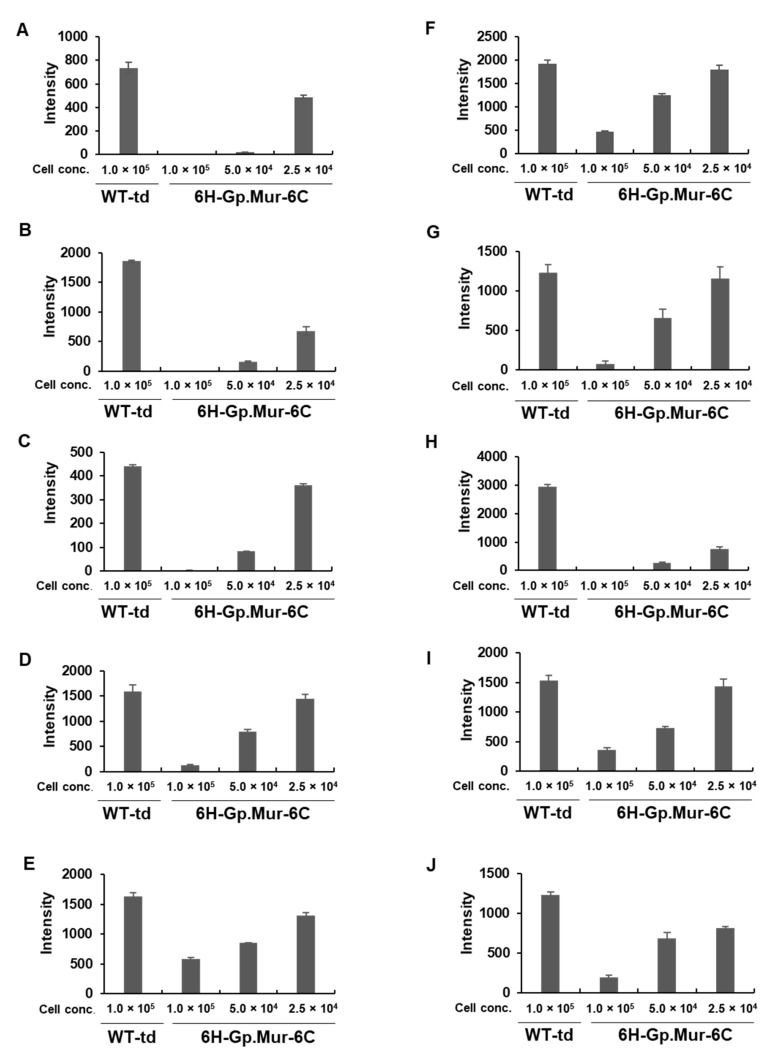
Quantification of hemagglutination by means of densitometry. Samples (**A**–**J**) were subjected to densitometric analysis. After grading the hemagglutination in samples according to the conventional tube method, the RBC agglutinates were carefully loaded onto 96-well plates and subjected to densitometric analysis. Letters correspond to the same samples used for the conventional tube method (Figure 3). X axis reflects the cell concentrations (cell conc.) of the adsorbing cells (WT-td and 6H-Gp.Mur-6C), while the Y axis reflects the signal intensity as measured by the densitometric analysis.

To better quantify the degree of hemagglutination inhibition by the adsorbing cells and given the dose–response effect we observed, we adopted a densitometric approach to quantitatively analyze hemagglutination in detail. We carefully pipetted the RBC agglutinates into 96-well plates and took images under microscopy (Supplementary Figure S2). The plates were then placed in an Amersham^TM^ Typhoon 5 Imager for densitometric scanning of the agglutinates. The scans were quantified using ImageQuant TL 8.2 software (Figure 4). Quantitative measurements of the intensity of the densitometric scans supported the existence of a dose–response inhibitory effect, with the highest insect cell concentrations resulting in the strongest inhibition (Figure 4 and Table 1).

#### 3.3.5. Comparison of the Grading Methods

These assays demonstrated that RBC hemagglutination was inhibited when anti-Gp.Mur antibody in the sera was adsorbed by insect cells expressing Gp.Mur antigen. At a cell concentration of 1.0 × 10^5^, all samples presented an agglutination grade of 0 to 1+, with an average % inhibition of 82%. At 5.0 × 10^4^ cells, sera presented agglutination grades of 0 to 3+ and an average % inhibition of 55%. At 2.5 × 10^4^ cells, agglutination grading ranged from 1+ to 4+, with an average % inhibition of 28%. When we assayed %inhibition more quantitatively by means of densitometric analysis, we recorded 89%, 64%, and 27% inhibition for insect cell concentrations of 1.0 × 10^5^, 5.0 × 10^4^, and 2.5 × 10^4^, respectively (Table 1). To compare these two grading approaches, we used a Pearson’s coefficient correlation (Pearson’s *r*) to assess their relationship. We observed moderate positive correlations (*r* = 0.63 for 1.0 × 10^5^, *r* = 0.67 for 5.0 × 10^4^, and *r* = 0.58 for 2.5 × 10^4^) between the two grading approaches, indicating that they were comparable. We compared the means of % inhibition generated according to the two grading approaches by means of paired Student *t*-test and the results were not significant (*p* > 0.05).

#### 3.3.6. Determining the Specificity of Gp.Mur Antigen Expressed in Insect Cells

**Figure 5:**
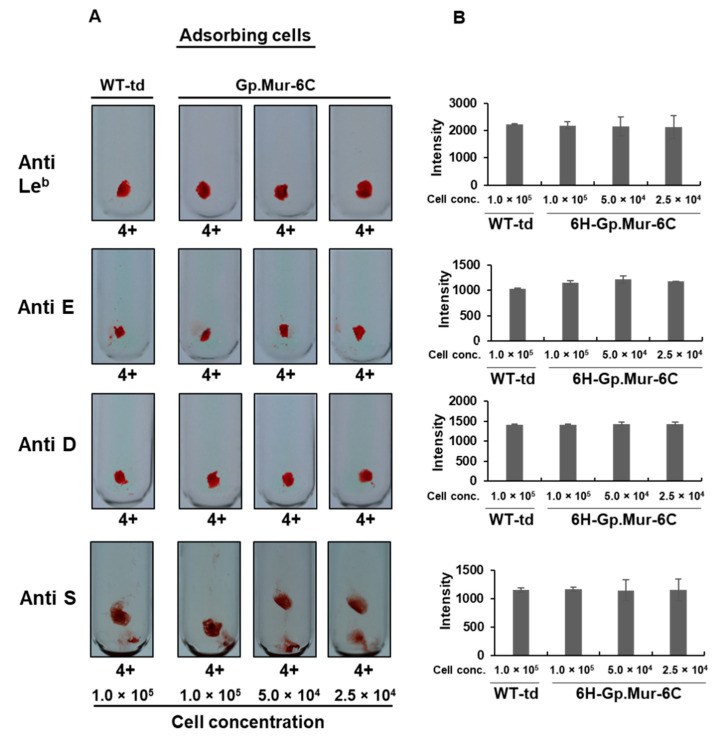
Hemagglutination inhibition by noncorresponding antibodies. Hemagglutination inhibition was performed on several antibodies not corresponding to Gp.Mur antigen, i.e., anti-Le^b^, anti-E, anti-D, and anti-S. (**A**) Visual grading from the conventional tube method. (**B**) Densitometric-based quantification of hemagglutination. On the X axis are the cell concentrations (cell conc.) of the adsorbing cells (WT-td or 6H-Gp.Mur-6C) and on the Y axis are the signal intensities of the RBC agglutinates.

Because there are numerous RBC antigens against which antibodies can develop, the binding specificity of recombinant RBC antigens to their corresponding antibodies is very important. In the above-described experiments, we have shown that anti-Gp.Mur antibody can recognize Gp.Mur antigen expressed in insect cells. We also wanted to determine if the Gp.Mur antigen expressed in insect cells binds nonspecifically to other RBC antibodies because such cross-reactivity could lead to inaccurate or confusing results with significant medical implications. To do that, we used serum samples containing antibodies from different blood groups, namely anti-Le^b^, anti-E, anti-D, and anti-S. The S antigen is highly homologous to the Gp.Mur antigen, so cross-reactivity with it may compromise test results if both anti-S and anti-Gp.Mur antibodies are present in a serum sample. We subjected these sera to the same procedures for antibody adsorption, grading, and quantification as conducted on sera preidentified as having anti-Gp.Mur antibody. Hemagglutination inhibition assays showed no observable or quantitative differences in levels of inhibition between WT-td-infected cells and v6H-Gp.Mur-6C-infected cells (Figure 5; Table 2), so our Gp.Mur antigen was highly specific.

#### 3.3.7. Stability of the Gp.Mur Antigen Expressed in Insect Cell

We wanted to assess if the Gp.Mur antigen expressed in insect cells is stable under conditions of long-term storage. For this experiment, we used freshly prepared suspensions of insect cells expressing Gp.Mur antigen, some of which was analyzed immediately and the remainder stored at 4 °C for 30 days and then subjected to the same analyses. There were no differences in the degree of hemagglutination inhibition between freshly prepared and stored samples (*p >* 0.05) (Supplementary Figure S3).

## 4. Discussion

In this study, we report a practical use of insect cell surface antigenic display for hemagglutination inhibition assay. We have demonstrated that insect cells displaying Gp.Mur antigen on their surfaces can substantially inhibit anti-Gp.Mur antibody in serum samples from patients preidentified as having that antibody. Moreover, as a significant advantage over the conventionally adopted tube method, we could quantitatively assess levels of hemagglutination inhibition by means of densitometry using our insect-cell-based system. Thus, our method can replace or supplement the conventional confirmatory method that involves using several RBC reagents.

We expressed Gp.Mur antigen on both Hi5 insect cells via recombinant baculovirus. We generated v6H-Gp.Mur-6C, which displayed a high level of surface expression of the antigen, demonstrating that the baculovirus 6C provides anchorage of the protein on insect cells. Baculovirus expression vector systems (BEVS) are superior to other expression systems such as yeast and bacteria as they exhibit better capacity for retaining post-translational modifications, protein oligomerization, and membrane protein expression [33]. Because our expressed recombinant protein was well recognized by the anti-Gp.Mur antibody in the human serum samples, as demonstrated by our hemagglutination inhibition assays, the recombinant Gp.Mur antigen is strongly expressed in insect cell membranes and with an appropriate antigenic configuration.

Although post-translational modifications (PTM) in insect cells differ slightly from mammalian cells, such as the inability of insect cells to produce end products containing terminal galactose and sialic acid [30], thousands of functional recombinant proteins, including human and animal-based vaccines, have been developed using BEVs [42]. Furthermore, based on our data, our recombinant Gp.Mur was able to maintain its antibody-binding capability as shown in our hemagglutination inhibition assay results.

The conventional method of RBC antibody identification makes use of a panel of phenotyped human RBCs. Because RBCs contain multiple antigens, a positive agglutination reaction means that antibodies exist in the sera against one or several antigens on the RBCs. As an indirect means to identify the antibody in a serum sample, a panel of human RBCs is typically used so that antigens from the RBCs that do not show an agglutination reaction with the serum can be ruled out. However, this method is ineffective in cases of autoantibodies, antibodies against high-frequency antigens, and antibodies that display a dosage effect. Given the limitations of this conventional identification system, a binary “yes or no” system has been proposed [18], which can only be achieved if the relevant RBC antigens are expressed individually. Previously, researchers have expressed and purified several of the relevant antigens using mammalian expression systems [20,21]. However, protein purification is tedious and requires up to 5 µg of the purified protein to inhibit the corresponding antibodies [19,23]. In the current study, we used a baculovirus system for high-level protein expression. Further development of our insect-cell-based system would circumvent the cost and effort associated with protein purifications. To the best of our knowledge, no insect cell-based hemagglutination inhibition assay for identifying human RBC antibodies has been reported previously.

Insect cell surface display of Gp.Mur antigen enabled specific recognition of the anti-Gp.Mur antibody in our patient serum samples. We also conducted hemagglutination inhibition assays on our recombinant baculovirus for other antibodies (anti-Le^b^, anti-E, anti-D, and anti-S), but the recombinant Gp.Mur antigen expressed in insect cells could not recognize those antibodies. Because S antigen shares conserved sequences with Gp.Mur antigen, the fact that our Gp.Mur antigen expressed in insect cells could not inhibit the hemagglutination activity of anti-S antibody strongly suggests that our recombinant Gp.Mur antigen is highly specific for anti-Gp.Mur antibody. Such specificity is highly important for identifying RBC antibodies as it informs which blood or blood products can be transfused into patients. Thus, the enhanced specificity of hemagglutination inhibition by recombinant RBC antigens could greatly aid in identifying the presence not only of the corresponding antibodies but also of antibodies of other specificities. Significantly, our recombinant Gp.Mur antigen expressed in insect cells is very stable, fully retaining its inhibitory characteristics even after storage at 4 °C for 30 days.

## 5. Conclusions

Anti-Gp.Mur antibody is one of the most commonly identified RBC antibodies in humans in Taiwan [6,10,43], and it may be highly prevalent in Southeast Asia, South Asia, and some parts of East Asia [7], although detailed prevalence and incidence studies on this antibody are lacking. This antibody has been implicated in hemolytic transfusion reactions and hemolytic disease of the fetus and newborn [14,15,16]. The technology that we have developed to express Gp.Mur antigen on insect cell surfaces by means of BEVS can be used to confirm and quantify anti-Gp.Mur antibody in human serum samples. The current approach for confirming anti-Gp.Mur antibody presence involves agglutinating a serum sample suspected of hosting anti-Gp.Mur antibody with another RBC reagent harboring Gp.Mur antigen. Hemagglutination inhibition using purified recombinant RBC antigen has been demonstrated to aid in the identification of the corresponding antibody as well as other nonspecific antibodies [19,21,23]. Our insect cell surface display technology for Gp.Mur antigen represents a convenient supplementary or replacement approach for the critical confirmation of anti-Gp.Mur antibody in human sera.

## Figures and Tables

**Table 1:** Comparison of hemagglutination inhibition using different concentrations of cells displaying Gp.Mur.

	RBC Agglutination (Preadsorption)		RBC Agglutination: Conventional Tube Method							RBC Agglutination: Densitometric Method						
			(Postadsorption)							(Postadsorption)						
Adsorbing Cells			WT-td	Gp.Mur-6C						WT-td	Gp.Mur-6C					
Cell Conc.			1.0 × 10^5^	1.0 × 10^5^		5.0 × 10^4^		2.5 × 10^4^		1.0 × 10^5^	1.0 × 10^5^		5.0 × 10^4^		2.5 × 10^4^	
Sample	IS ^a^	AHG ^b^	Grade	Grade	% inh ^c^	Grade	% inh	Grade	% inh	Int ^d^	Int	% inh	Int	% inh	Int	% inh
A	3+	1+	3+	0	100	1+	67	2+	33	735	0	100	19	97	486	34
B	4+	3+	4+	1+	75	2+	50	2+	50	1858	0	100	163	91	678	64
C	4+	2+	3+	0	100	0	100	1+	67	442	1	99	82	81	359	19
D	3+	1+	4+	1+	75	2+	50	4+	0	1584	124	92	796	50	1444	9
E	4+	3+	4+	1+	75	3+	25	4+	0	1628	583	64	844	48	1312	19
F	4+	1+	3+	1+	67	2+	33	3+	0	1919	460	76	1245	35	1788	7
G	3+	3+	4+	1+	75	2+	50	3+	25	1232	79	94	660	46	1155	6
H	4+	1+	4+	0	100	1+	75	2+	50	2953	0	100	264	91	747	75
I	4+	1+	4+	1+	75	2+	50	3+	25	1538	361	77	730	53	1434	7
J	2+	3+	4+	1+	75	2+	50	3+	25	1228	198	84	687	44	812	34
Average					82		55		28			89		64		27

^a^ Immediate spin phase; ^b^ antihuman globulin phase; ^c^ % inhibition; ^d^ signal intensity.

**Table 2:** Summary of hemagglutination inhibition for nonspecific antibodies.

	RBC Agglutination: Conventional Tube Method							RBC Agglutination: Densitometric Method						
Adsorbing Cells	WT-td	Gp.Mur-6C						WT-td	Gp.Mur-6C					
Cell Conc.	1.0 × 10^5^	1.0 × 10^5^		5.0 × 10^4^		2.5 × 10^4^		1.0 × 10^5^	1.0 × 10^5^		5.0 × 10^4^		2.5 × 10^4^	
Sample	Grade	Grade	% inh ^a^	Grade	% inh	Grade	% inh	Int ^b^	Int	% inh	OD	% inh	OD	% inh
Anti-Le ^b^	4+	4+	0	4+	0	4+	0	2222	2180	2	2147	3	2125	4
Anti-E	4+	4+	0	4+	0	4+	0	1427	1428	0	1395	2	1399	2
Anti-D	4+	4+	0	4+	0	4+	0	1046	1191	0	1144	0	1184	0
Anti S	4+	4+	0	4+	0	4+	0	1190	1365	0	1216	0	1181	0.70

^a^ % inhibition; ^b^ signal intensity.

## Data Availability

Data supporting reported results are available from the corresponding author upon request.

## Supplementary Materials

The following are available online at https://www.mdpi.com/article/10.3390/diagnostics11060966/s1, Figure S1: Development of an insect cell-based hemagglutination inhibition assay using Gp.Mur antigen expressed on insect cells, Figure S2: Microscopic visualization of hemagglutination inhibition, Figure S3: Stability of the Gp.Mur antigen expressed on Hi5 cells upon storage at 4 °C for 30 days.
