# Supplementary material for: Identification and Quantification of Anti-Gp.Mur Antibodies in Human Serum Using an Insect-Cell-Based System
Source: Diagnostics (Basel). 2021 May 27;11(6):966. doi: 10.3390/diagnostics11060966 (PMC8227843; doi:10.3390/diagnostics11060966)
Supplement: Supplementary file 1 [file diagnostics-11-00966-s001.zip › diagnostics-1170571-SI.pdf]

Identification and Quantification of Anti-Gp.Mur Antibodies in Human Serum Using an Insect Cell-based System

Supplementary data

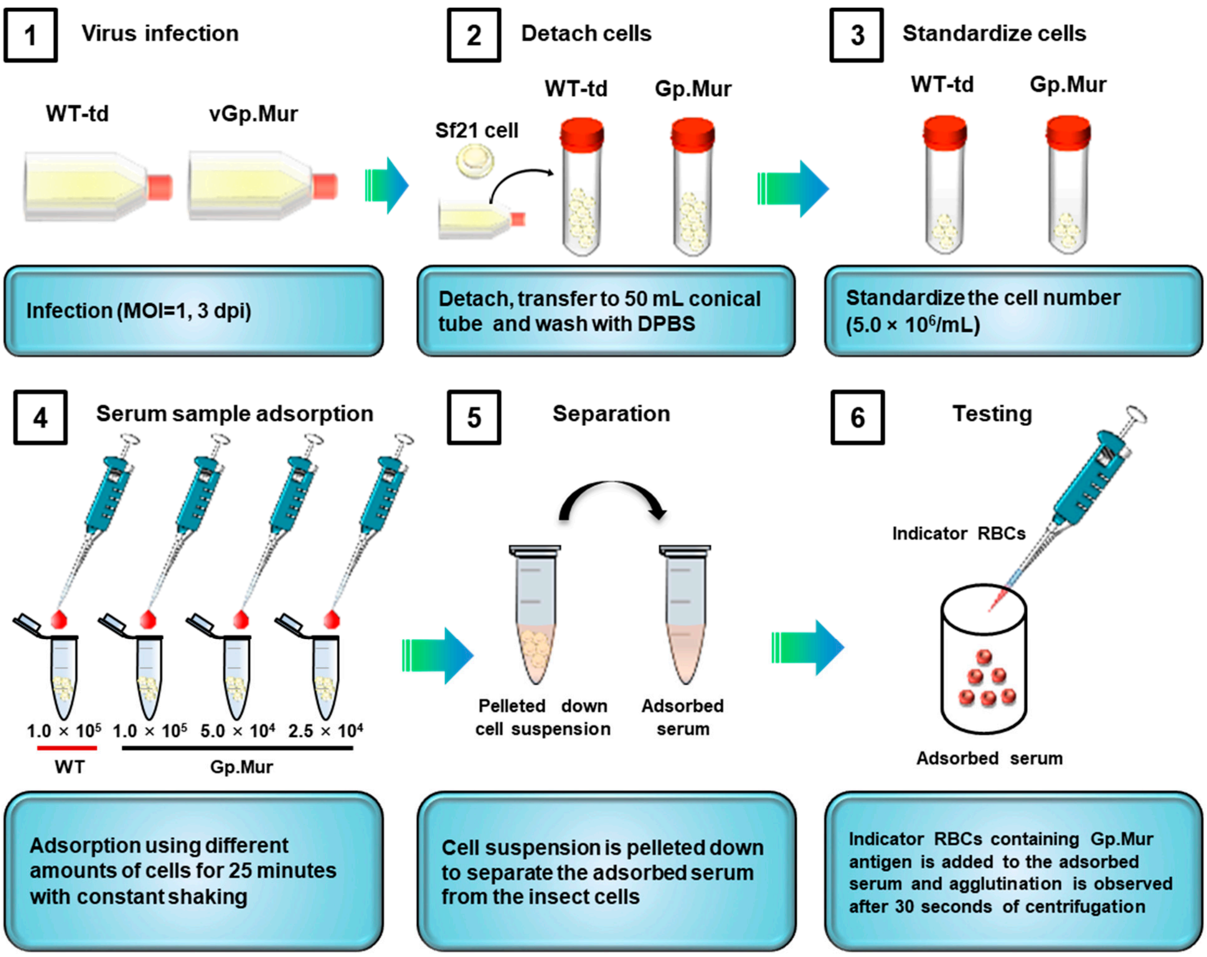

Supplementary Figure S1. Development of an insect cell-based hemagglutination inhibition assay using Gp.Mur antigen expressed on insect cells. 1) Insect cells are infected either with Gp.Mur recombinant baculovirus or wild type baculovirus with the red fluorescence only (WT-td) at MOI=1. 2) After 3 days post infection (dpi), the cells are detached from the flasks and washed with DPBS. 3) Cell concentrations are standardized in a cell suspension using DPBS (5.0 × 10^6/mL). 4) Varying amounts of cell suspension are used to adsorb the serum samples with preidentified anti-Gp.Mur by mixing the cell suspension with the serum under constant agitation for 25 min. 5) The insect cells are separated from the cell suspension by centrifugation and the adsorbed serum is transferred to a separate test tube. 6) Indicator RBCs expressing Gp.Mur antigen are added to the adsorbed serum to confirm anti-Gp.Mur antibody presence.

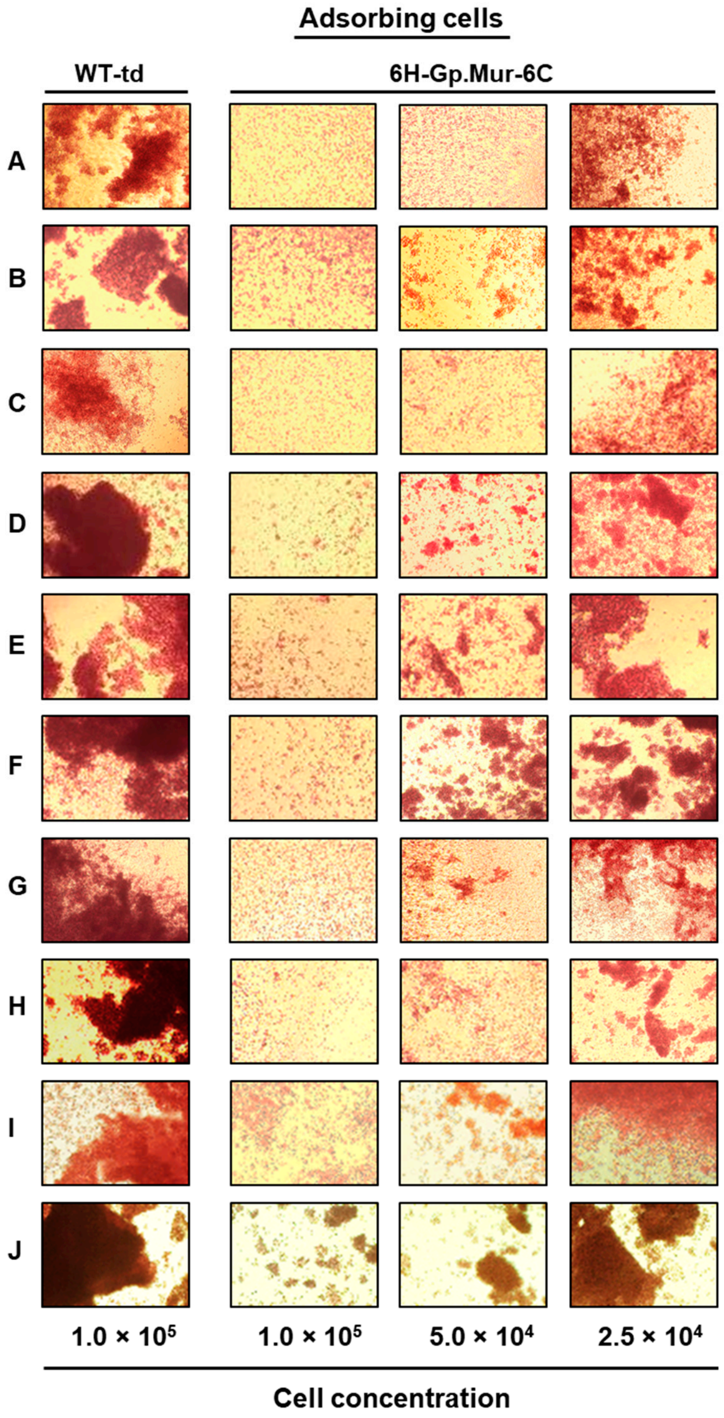

Supplementary Figure S2. Microscopic visualization of hemagglutination inhibition. Samples (**A-J**), after visual analysis of hemagglutination, were carefully pipetted onto 96-well plates and the agglutination was observed at 10× magnification. The letters correspond to the same samples in Figure 3 and Figure 4.

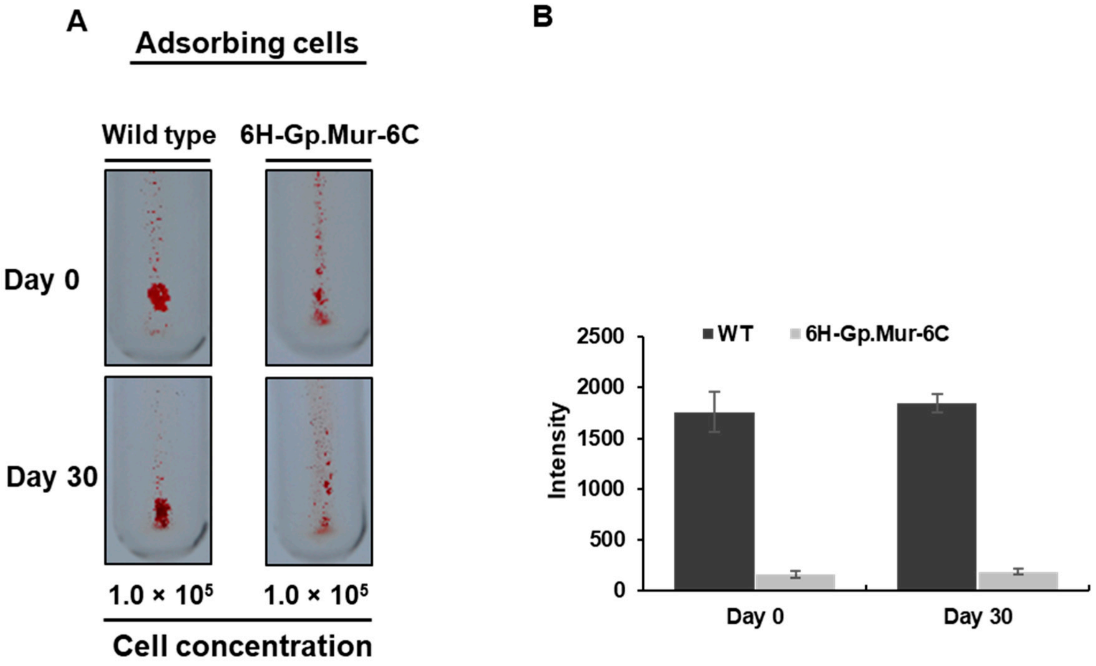

Supplementary Figure S3. Stability of the Gp.Mur antigen expressed on Hi5 cells upon storage at 4 °C for 30 days. Comparison of hemagglutination inhibition ability for freshly-prepared insect cells expressing Gp.Mur antigen (day 0) and the same cells stored at 4 °C for 30 days (day 30), based on the conventional tube method (**A**) and densitometric quantification (**B**).
